# Single-cell RNA profiling of colorectal granular-type laterally spreading tumor uncovers progression trajectory toward carcinoma and transcriptional signatures favoring lateral morphogenesis

**DOI:** 10.3389/fonc.2025.1552841

**Published:** 2025-09-12

**Authors:** Yueqing Gong, Yuxin Zhang, Xun Liu, Rongli Cui, Jingjing Lu, Jun Li, Fang Gu, Jing Zhang, Shigang Ding, Weiwei Fu

**Affiliations:** ^1^ Department of Gastroenterology, Peking University Third Hospital, Beijing, China; ^2^ Department of Gastroenterology of Peking University Third Hospital, Beijing Key Laboratory for Helicobacter Pylori Infection and Upper Gastrointestinal Diseases (BZ0371), Beijing, China

**Keywords:** laterally spreading tumor (LST), adenomatous granular-type LST, single-cell transcriptome, metaplasia, morphogenesis, colorectal precancerous lesion, adenoma

## Abstract

**Background:**

Colorectal laterally spreading tumors (LSTs) are defined as non-protruding neoplasms exceeding 10 mm in diameter that grow primarily along the intestinal wall. The morphogenetic mechanisms and evolutionary trajectories of granular-type LSTs (LST-Gs) towards colorectal carcinoma remain unclear.

**Methods:**

In this study, we investigate the transcriptional features of LST-Gs using single-cell RNA sequencing technology by comparing them with protruded-type adenomas (PAs) and normal mucosal tissues.

**Results:**

Adenomatous LST-Gs harbor an epithelial cell population with metaplastic differentiation, which are almost absent in PAs. Cells with a high degree of differentiation in LST-G demonstrate enhanced immunogenicity and robust adhesion/junction interactions. Furthermore, LST-Gs show upregulated expression of molecular chaperones and metallothioneins compared to PAs, reflecting a more hostile microenvironment similar to that observed in carcinoma stages. These characteristics suggest that LST-G exhibits greater heterogeneity compared to the earliest colorectal adenomas, mirroring the progression from precancerous states to cancer. Notably, the Arp2/3 complex is significantly upregulated in highly differentiated LST-G cell populations, potentially facilitating cell migration along the basement membrane, which highlights the similarities in cell motility between adenomatous LST-G and normal mucosal epithelium as well as serrated polyps (SERs).

**Conclusion:**

The differentiation state of cells within LST-G exhibits a close correlation with their diverse characteristics. Metaplastic differentiation, as a prominent feature at the transcriptional level, demonstrates significant associations with the genomic features, morphogenesis, and tumor progression of LST-G.

## 1 Introduction

Timely endoscopic resection of colorectal polyps is crucial for mitigating the risk of colorectal cancer (CRC). Smaller polyps, less than 10 mm in diameter, rarely harbor malignancy and can be efficiently removed via endoscopic techniques. Laterally spreading tumors (LSTs), defined as non-protruding neoplasms exceeding 10 mm in diameter that grow primarily along the intestinal wall (1), present a more complex scenario. Specifically, larger LSTs, exceeding 20 mm, carry a substantially elevated risk of submucosal invasive cancer (2). Troublingly, the endoscopic management of these lesions is technically demanding, often culminating in incomplete resection and local recurrence (3, 4).

Based on their endoscopic morphology, LSTs are categorized into two distinct types: LST-granular (LST-G), characterized by even or uneven surface nodules, and LST-nongranular (LST-NG), distinguished by a smooth surface. Significantly, the KRAS mutation frequency is markedly higher in LST-G adenomas relative to protruded adenomas (PAs), whereas it is diminished in LST-NG adenomas (5–7). Until now, the relationship between the molecular characteristics of LSTs and their progression to carcinoma remains unclear. What is established is that specific molecular features of adenomatous LST-NG are more closely linked to cancer (8). Adenomatous LST-NG exhibits stronger WNT signaling pathway activity, and displays higher p53 activity, Ki-67 proportion, microvascular density, fibrosis, inducible nitric oxide synthase (iNOS), and nitrotyrosine (NT) levels compared to adenomatous LST-G, while expressing lower levels of acidic mucin. Notably, within LST-NG subtypes, only the pseudodepressed variant (LST-NG-PD) demonstrates significant submucosal invasion rates (~40%), while the flat elevated type (LST-NG-F) rarely progresses to malignancy. In contrast, LST-G adenomas appear to follow a more conventional adenoma-carcinoma sequence. These lesions typically accumulate gain-of-function mutations in oncogenes (particularly KRAS) and loss-of-function mutations in tumor suppressor genes (e.g., TP53), ultimately developing into microsatellite stable (MSS) tumors (9). The elevated KRAS mutation rate in LST-G suggests these lesions may represent a more advanced stage in tumorigenesis, consistent with their observed higher rates of submucosal invasion. Nevertheless, it remains unclear whether the molecular evolution of adenomatous LST-G to carcinoma fundamentally differs from that of protruded-type adenomas.

A recent single-cell RNA study (Colorectal Molecular Atlas Project, COLON MAP) shows that the molecular characteristics of the colorectal precancerous lesions, as manifested by their pathological phenotypes, may also affect their morphogenesis (9). These precancerous lesions can be broadly classified into adenoma (AD) and serrated polyps (SER) based on their pathological nature. AD-specific cells (ASC) express genes associated with WNT signaling pathway activation and exhibit stronger stemness than normal tissue stem cells, while serrated-specific cells (SSC) do not demonstrate WNT signaling pathway activation or robust stemness but show a higher degree of differentiation. SSCs express genes typically associated with gastric epithelial cells, such as MUC5AC, AQP5, and MUC17, suggesting a metaplastic origin. Interestingly, AD and SER exhibit distinct morphological features: SERs are often flat or sessile, while protruded-type SERs are infrequent; ADs are primarily protruded and sessile, with flat lesions being the minority (10–12). Thus, the morphology of these precancerous lesions may be associated with cells differentiation status.

Generally, tissue morphogenesis is closely linked to cell movement. In normal intestinal mucosal epithelium, differentiated cells undergo collective migration along the basement membrane, and this migration is not driven by a pushing force resulting from cell division (i.e., mitotic pressure) (13). Recent studies reveal that, mediated by the Arp2/3 complex, enterocytes possess small, F-actin-rich basal feet that contact the basement membrane, orienting in the direction of cell movement. Through actin polymerization, these structures, akin to lamellipodia, generate protrusive forces, facilitating active cell migration along the basement membrane (13). Though the migratory mechanism of normal intestinal mucosal epithelial cells is well characterized, the morphogenesis of LSTs remains unclear. The correlation between the molecular characteristics of LST-Gs and their morphology requires further exploration.

In this study, we further unravel the mechanisms underlying malignant transformation and morphogenesis of adenomatous LST-G using single-cell transcriptome technology. Adenomatous LST-G is examined and compared with protruded-type AD and normal mucosal tissue to elucidate the transcriptional features in LST-G and clarify their associations with tumor progression and morphogenesis. This will shed light on the malignant progression and microenvironment of LST-G, and provide a basis for precision research into pathogenesis and prevention of CRC.

## 2 Materials and methods

### 2.1 Participants

Seven patients diagnosed with adenomatous LST-G or PA were enrolled from Peking University Third Hospital, China. The study protocol was approved by the Medical Research Ethics Committee of Peking University Third Hospital. All patients were diagnosed based on endoscopic and histological examination results. Patients with other infections, gastrointestinal diseases, or other tumors were excluded. Six LST-G samples, two normal mucosa (NL) samples, and two PA samples was incorporated into our single-cell RNA analysis (**Table 1**).

**Table 1:** Clinical characteristics of each sample used in this scRNA-seq study.

Sample Name	Patient	Gender	Endoscopic morphology	Pathology^1^	Location
P1_L	P1	M	LST-G	TA	Rectum
P1_N	P1	M	NL	Normal	Rectum
P2_L	P2	M	LST-G	TVA	Transverse colon
P3_L	P3	M	LST-G	TVA	Caecum
P3_P	P3	M	PA	TA	Sigmoid
P4_L	P4	F	LST-G	TA	Rectum
P5_N	P5	M	NL	Normal	Caecum
P5_L	P5	M	LST-G	TA	Caecum
P6_L	P6	M	LST-G	TVA	Sigmoid
P7_P	P7	F	PA	TA	Sigmoid

^1^ TA, tubular adenoma; TVA, tubulovillous adenoma.

### 2.2 Sample collection and processing

Tissue dissociation was conducted using a Tumor Dissociation Kit (Miltenyi Biotec) according to the manufacturer’s instructions. Fresh resected tissues were cut into 1 mm pieces and transferred to C tubes containing an enzyme mix (enzymes H, R, and A in RPMI 1640 medium). The gentleMACS program (37C_h_TDK_2) was run in a MACSmix Tube Rotator (Miltenyi). The resulting suspension was mixed with 10 ml of ice-cold RPMI 1640 medium and filtered through a 70-µm tip strainer (BD Falcon). The suspension was then centrifuged at 300g for 5 minutes at 4°C. After removing the supernatant, the pellet was resuspended in 1 ml of red blood cell lysis buffer and incubated at room temperature for 5 minutes. The suspension was again centrifuged at 150g for 5 minutes at 4°C, and the supernatant was removed. The pellet was resuspended in 0.04% bovine serum albumin (BSA)/D-PBS.

### 2.3 Library preparation and sequencing

Library preparation was performed according to the instructions in the 10X Chromium Single Cell 3’ v3 kit. The libraries were then pooled and sequenced on the Illumina NovaSeq 6000 system. Raw data was deposited in a publicly available database.

### 2.4 Quality control and data pre-processing

The sequencing data from 10x Genomics were aligned and quantified using the CellRanger software package (version 6.0.0) against the human reference genome (hg38). Gene expression matrices were imported and processed using the Seurat R package (version 4.1.0) (14). Low-quality cells, characterized by expressing fewer than 1000 or more than 8000 genes, or having over 50% of unique molecular identifiers (UMIs) mapping to mitochondrial genes, were excluded. Considering the rapid renewal and active respiratory metabolism of gastrointestinal mucosal epithelial cells, we selected a high threshold for mitochondrial counts. Such high mitochondrial thresholds have also been applied in previous single-cell transcriptome analyses of gastrointestinal mucosal epithelial cells (15). Following filtering, the remaining cells underwent routine clustering, dimensionality reduction, or other downstream analysis procedures.

### 2.5 Acquisition and processing of public datasets

We obtained level 3 single-cell transcriptome expression data and metadata from the Colorectal Molecular Atlas Project (COLON MAP) (9) through the Human Tumor Atlas Network (HTAN) Data Portal (https://humantumoratlas.org/). Additionally, scRNA-seq expression matrices and metadata for the SMC and KUL3 cohorts were obtained from the NCBI Gene Expression Omnibus (GEO) database under the accession codes GSE132465 and GSE144735 (16). For these single-cell transcriptome datasets, we mainly followed the procedures outlined in the source paper. Briefly, after quality control to remove low-quality cells, the COLON MAP dataset was processed through the single-cell regulatory network inference and clustering (SCENIC) pipeline to generate a regulon activity enrichment matrix (17). Based on this matrix, dimensionality reduction and clustering were performed, yielding results consistent with the source paper. The identified epithelial cell subpopulations included typical populations found in normal mucosa, as well as ASC and SSC populations uniquely present in AD and SER, respectively. For the KUL3 and SMC3 datasets, we followed the standard Seurat processing pipeline to identify epithelial cell populations from different sample types. Furthermore, bulk transcriptome data from the TCGA-COAD and TCGA-READ cohorts were obtained using the TCGAbiolinks R package (18).

### 2.6 Integration of single-cell transcriptome data

We integrated the COLON MAP dataset with our LST dataset within the batch-corrected RNA space, following standard integration methods in the Seurat package v3/v4. Briefly, we treated our LST dataset and the COLON MAP dataset as two batches and used the FindIntegrationAnchors and IntegrateData functions in the Seurat package for integration.

### 2.7 Regulon network prediction and regulon activity calculation

We used the SCENIC pipeline to predict the transcriptional regulatory network and calculate regulon activity, following methods outlined in previous literature (9). Briefly, for our LST dataset, we merged data from all samples to obtain a combined expression matrix, which was then converted into a loom file and processed through the pySCENIC pipeline (version 0.11). The motif-to-TF annotations database used in the analysis was “motifs-v9-nr.hgnc-m0.001-o0.0.tbl”. cisTarget was performed using default parameters and two hg38.feather ranking databases, including “hg38:refseq-r80:500bp_up_and_100bp_down_tss.mc9nr.feather” and “hg38:refseq-r80:10kb_up_and_down_tss.mc9nr.feather”. After analysis, the regulon activity matrix obtained from LST datasets was used for clustering/dimensionality reduction to identify cell populations. Additionally, to reproduce the cell population annotations of the COLON MAP dataset, we also analyzed this dataset using the SCENIC pipeline, with parameters and databases following the source paper.

### 2.8 Identification and scoring of cell populations

We first identified epithelial cell subpopulations in the COLON MAP dataset according to the scheme outlined in the source paper (9). Briefly, we identified cell populations based on the expression of typical marker genes (**Supplementary Table S1**), the proportion of each subpopulation in various sample types, and the distribution of subpopulations in Uniform Manifold Approximation and Projection (UMAP) based on regulon space. For our LST dataset, to more accurately determine the nature of the populations, we integrated it with the COLON MAP dataset and used the cell population annotations from the COLON MAP dataset as a reference to identify populations in the LST dataset. Furthermore, we verified the annotations of the populations through the expression of typical marker genes. Finally, the identified populations were projected onto a UMAP based on the regulon space of the LST dataset for visualization.

Additionally, we scored cell populations using multiple signatures to further determine their nature. Among these signatures, the signatures for metaplasia, fetal genes, and the Wnt signaling were derived from the COLON MAP literature (**Supplementary Table S2**). Furthermore, using the COSG R package (19) (parameter: mu = 200), we identified top marker genes for ASC, SSC and absorptive cell (ABS) populations in the COLON MAP dataset. This approach leverages cosine similarity to assess gene expression specificity, and the resulting top marker genes were selected as signatures for scoring (**Supplementary Table S2**). Additionally, we sourced gene sets from various curated databases, including Gene Ontology (GO) (20), Kyoto Encyclopedia of Genes and Genomes (KEGG) (21), Reactome (22), and the Molecular Signatures Database (MSigDB) Hallmark collection (23). Scores were calculated using the AddModuleScore function in the Seurat package.

### 2.9 Inference of cell-cell communication

The CellChat R package (Version 1.5.0) was used for the inference and visualization of intercellular communication, following the standard workflow of the software (24). Briefly, the normalized expression matrix from Seurat and cell population annotations were input into the CellChat pipeline. Downstream analysis included inferring cell-cell communication probability (computeCommunProb function) at both signaling pathway and gene levels. Significant signaling pathways were visualized as circle plots (netVisual_aggregate function) and heatmaps (netVisual_heatmap function). Ligand-receptor pairs were visualized as bubble plots (netVisual_bubble function).

### 2.10 Identification of differentially expressed genes and enrichment analysis

Differentially expressed genes were identified using the FindMarkers function in Seurat. Additionally, after ranking genes based on the logFC of their expression levels, we conducted gene set enrichment analysis (GSEA) using the ClusterProfiler package (version 4.2.2) (25). The gene sets used were derived from GO, KEGG, Reactome, and MSigDB.

For bulk RNA-seq data from TCGA, single-sample gene set enrichment analysis (ssGSEA) (26) was used to calculate the scores for the chaperone signature (**Supplementary Table S2**). We then calculated the Spearman correlation coefficient between this score and the expression of all genes in the transcriptome data. Based on this coefficient, genes were ranked and GSEA was performed.

### 2.11 Pseudotime analysis

Due to the strong heterogeneity of samples in RNA space, to better describe differentiation states using pseudotime, we established pseudotime based on regulon space. Specifically, the regulon activity matrix was input into the Scanpy pipeline (version 1.9.1) (27), and diffusion pseudotime was calculated using the “sc.tl.dpt” function for the pseudotime heatmap [“plot_pseudotime_heatmap” function in the Monocle R package (28)].

### 2.12 Visualization

In addition to the visualization functions in the tools mentioned above, some results were visualized using ggplot2. Additionally, we used ComplexHeatmap (version 2.10.0) (29) for heatmap generation and Cytoscape (version 3.9.1) (30) for network visualization.

### 2.13 Statistical methods for comparison

Wilcoxon test was utilized for the comparison between two groups. For comparisons involving multiple groups, Kruskal-Wallis test was employed, subsequently complemented by Dunn’s test for comparisons between group pairs, using FDR for p-value adjustment.

## 3 Results

### 3.1 Adenomatous LST-G harbors cell population with metaplastic differentiation

In order to investigate the transcriptional features of adenomatous LST-G, we incorporated six LST-G samples, two NL samples, and two PA samples into our single-cell RNA analysis (**Supplementary Figure 1A** and **Table 1**). Notably, all LST-G and PA lesions underwent rigorous clinicopathological examination, confirming their pathological nature as adenoma. The specimens were all endoscopically obtained and processed by expert pathologists, with patients having received no prior treatment before endoscopy. Following quality control measures, 31,213 cells were retained from the sequencing data. These cells were subsequently classified into seven major cell lineages based on the expression patterns of conventional marker genes (**Supplementary Figures 1B, C**). Next, we employed clustering and dimensionality reduction techniques on the epithelial lineage to identify distinct cell subpopulations. However, akin to previous studies (9), we observed significant heterogeneity among cells derived from different samples within the RNA space (depicted in **Supplementary Figure 1D**), which could potentially obscure shared characteristics across diverse sample sources.

**Figure 1:**
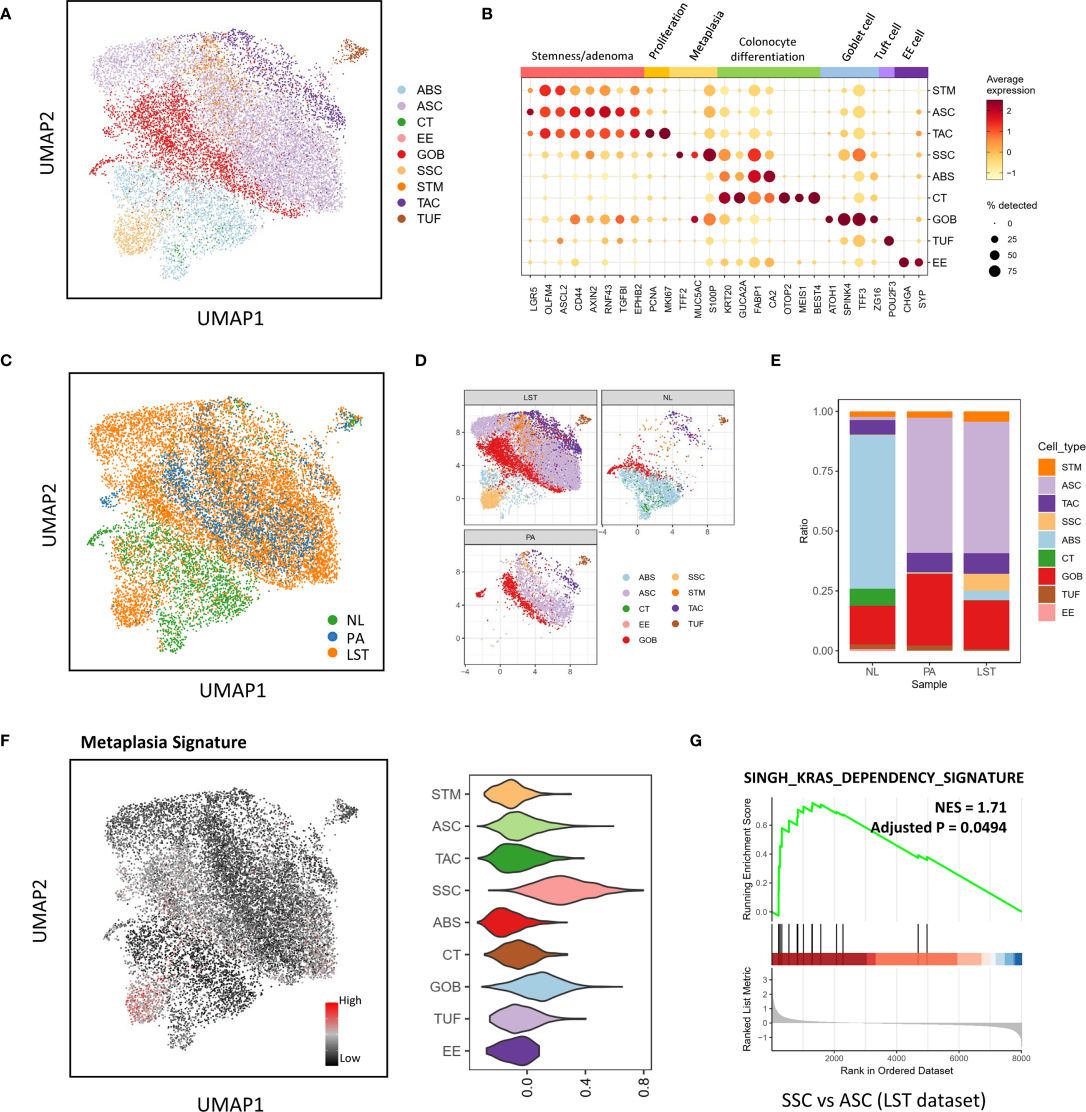
Cell atlas of granular-type laterally spreading tumors (LSTs). **(A)** UMAP plot based on regulon space illustrating the epithelial cell subpopulations. ABS, absorptive cells; ASC, adenoma-specific cells; CT, top colonocytes; EE, enteroendocrine cells; GOB, goblet cells; STM, stem cells; SSC, serrated specific cells; TAC, transit amplifying cells; TUF, tuft cells. **(B)** Expression profiles of key marker genes within distinct epithelial cell subpopulations. **(C–E)** Comparison of the proportions of epithelial subpopulations present in the LST, protruded adenoma (PA), and normal mucosa (NL) groups. **(F)** UMAP plot and violin plot illustrating the metaplasia signatures of epithelial populations. **(G)** Gene Set Enrichment Analysis (GSEA) outcomes, comparing the SSC and ASC populations.

To address this issue, we conducted SCENIC analysis to reduce dimensionality using regulon space. To enhance the accuracy of cell population classification and explore potential correlations with existing research, we also integrated the COLON MAP dataset with our LST dataset within the batch-corrected RNA space. By utilizing the COLON MAP dataset as a reference, we were able to precisely identify various cell types. The results showed that the UMAP based on regulon space effectively adjusted for polyp-specific effects (**Supplementary Figure 1E**). The cell populations identified using the reference dataset (presented in **Figure 1A**, **Supplementary Figures 1F, G**) were in agreement with the expression of conventional marker genes (**Figure 1B**, **Supplementary Figure 2A** and **Supplementary Table S1**). These populations encompassed a diverse array of cells characteristic of normal intestinal mucosal epithelium, including absorptive cells (ABSs), crypt top colonocytes (CTs), enteroendocrine cells (EEs), goblet cells (GOBs), stem cells (STMs), transit amplifying cells (TACs), and tuft cells (TUFs).

Notably, the most prevalent cell population in both PA and LST samples was virtually absent in NL samples (as shown in **Figures 1C–E**). This particular population exhibited high stemness and gene expression profiles similar to ASCs in the COLON MAP dataset (**Supplementary Figure 2B, C**). Due to their close resemblance to ASCs, we referred to these cells as ASCs. Intriguingly, despite the pathological nature of LST samples being adenoma, we identified a population with similarities to SSCs in the COLON MAP dataset, which exhibited high metaplasia and fetal signature scores (**Figure 1F**, **Supplementary Figures 2D, E**). Consequently, we also designated this population as SSC. SSC cells were scarcely detectable in AD samples from the COLON MAP dataset (**Supplementary Figure 2F**). However, in our adenomatous LST samples, a significant number of SSCs and ABSs were present (**Figure 1E** and **Supplementary Figure 2G**). By comparison, in PA samples, there were almost no SSCs or ABSs. In short, compared to PA, LST contained a higher proportion of more differentiated cell populations, like SSCs or ABSs.

Further GSEA analysis exhibited an elevated expression level of a KRAS dependency signature (SINGH_KRAS_DEPENDENCY_SIGNATURE in MSigDB) (31) in SSC relative to ASC cells (**Figure 1G**). This signature was derived from the cells that exhibit high dependency on mutated KRAS. This finding was consistent with the fact that LST-G exhibits a higher frequency of KRAS mutations.

### 3.2 SSC and ABS cell populations in LST-G exhibit strengthened immunogenicity, adhesion/junction and EGFR signaling communication

**Figure 2:**
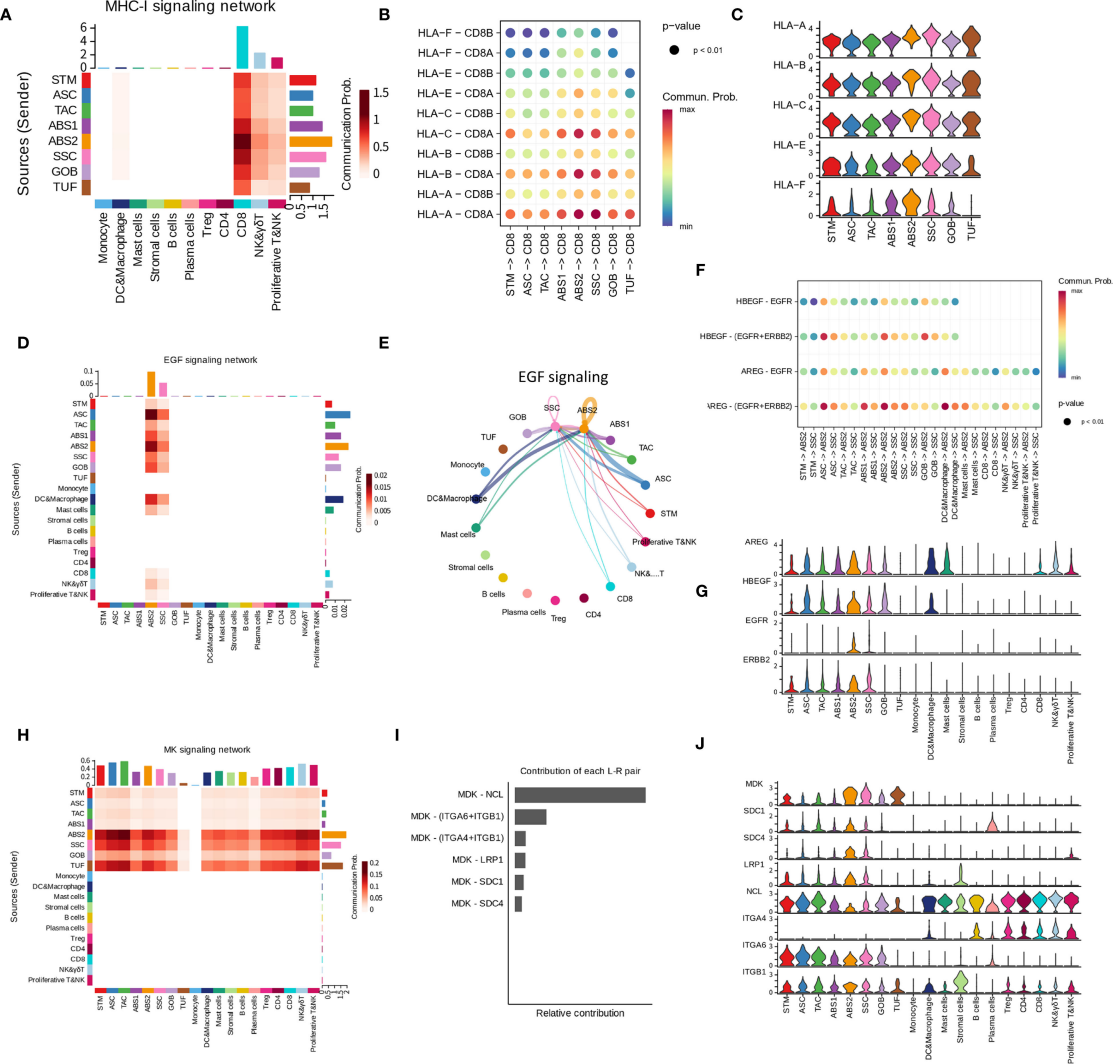
Intercellular interactions associated with key pathways in LST-Gs. **(A)** Heatmap illustrating the strength of intercellular interactions in MHC-I signaling network. Row names are senders, and column names are receivers. The right legend, “communication prob”, indicates the strength of communication. **(B)** Bubble plot illustrating the significant interactions (L-R pairs) of MHC-I signaling from epithelial subpopulations to CD8 cells. **(C)** Expression of genes contributing to the interactions in MHC-I signaling network. **(D)** Heatmap illustrating the strength of communications via EGFR signaling. **(E)** Circle plot showing the communications via EGFR signaling. **(F)** Bubble plot illustrating the significant interactions (L-R pairs) of EGFR signaling. **(G)** Expression of genes contributing to the interactions in EGFR signaling network. **(H–J)** Intercellular communication status via MK signaling. **(I)** indicates the contributions of L-R pairs in the signaling network.

The heterogeneity of cell populations due to differentiation in LSTs may alter cell communication status. Thus, we further conducted a cell-cell communication analysis. CellChat results revealed that the communication status between epithelial cells and immune cells in LSTs was associated with the epithelial differentiation levels. To highlight the influence of cell differentiation on communication status, we stratified the continuous ABS population into two distinct groups according to their differentiation levels: ABS1, characterized by relatively lower differentiation, and ABS2, exhibiting relatively higher differentiation. Firstly, compared to the ASC population, the highly differentiated SSC and ABS populations exhibited strengthened communications with CD8, NK, and γδT cells via MHC-I signaling (**Figures 2A, B**). This augmentation can be attributed to the elevated expression of MHC-I molecules in SSC, ABS1 and ABS2 (**Figure 2C**).

Moreover, CellChat results also exhibited other interaction pairs between differentiated populations and immune cells. SSC and ABS2 populations engaged in communication with Treg, CD8, and proliferative T&NK populations via JAM1-integrin αLβ2 interactions, while the ASC population did not exhibit significant activity in this pathway (**Supplementary Figures 3A–C**). Furthermore, when compared to ASC, SSC and ABS2 populations demonstrated stronger BAG6-NCR3 interactions with proliferative T&NK population (**Supplementary Figures 3D–F**). Additionally, ABS2 populations exhibited higher expression levels of IFNGR1 and IFNGR2 relative to other epithelial cell populations, rendering them more responsive to IFNγ regulation (**Supplementary Figures 3G–I**). Moreover, ABS1 and ABS2 populations displayed elevated expression of galectin-9 (LGALS9), which has the potential to interact with immune cell receptors such as CD45, TIM-3 (HAVCR2), and CD44 (**Supplementary Figures 3J–L**). All these communications can modulate the function of immune populations (32–34).

In addition to these immune-related interactions, a series of observations also highlighted the characteristics of highly differentiated epithelial cells in cell adhesion and junctions (**Supplementary Figure 4**). When compared to ASC, ABS and SSC populations demonstrated upregulated expression of E-cadherin (CDH1), claudin-3 (CLDN3), occludin (OCLN), desmocollin-2 (DSC2), desmoglein-2 (DSG2), and CEACAMs, leading to enhanced cell-cell adhesion and junction formation (**Supplementary Figures 4A, E,G**). Furthermore, laminins, crucial components of the basement membrane, were upregulated in ABS2 and SSC populations, indicating a stronger capacity of these populations to participate in the maintenance of basement membrane structure (**Supplementary Figures 4F, H**). ABS2 and SSC populations also exhibited high levels of integrins, enabling them to establish firmer connections with the basement membrane through interactions with laminins (**Supplementary Figure 4I**).

Notably, within LSTs, only ABS2 and SSC populations exhibited EGFR signaling-related communication (**Figures 2D–F**). These populations displayed significantly higher expression of EGFR and ERBB2 compared to other epithelial cell populations (**Figure 2G**). It is worth mentioning that no EGFR-related communication was detected between populations in PA samples. Furthermore, all epithelial cell populations were capable of communicating with nearly all cell populations through midkine (MDK) (**Figures 2H–I**). ABS2 and SSC populations exhibited significantly higher expression of MDK compared to less differentiated populations such as ASC, resulting in stronger interactions (**Figure 2J**).

### 3.3 Transcriptional signatures of epithelial cell subpopulations vary across distinct sample types

**Figure 3:**
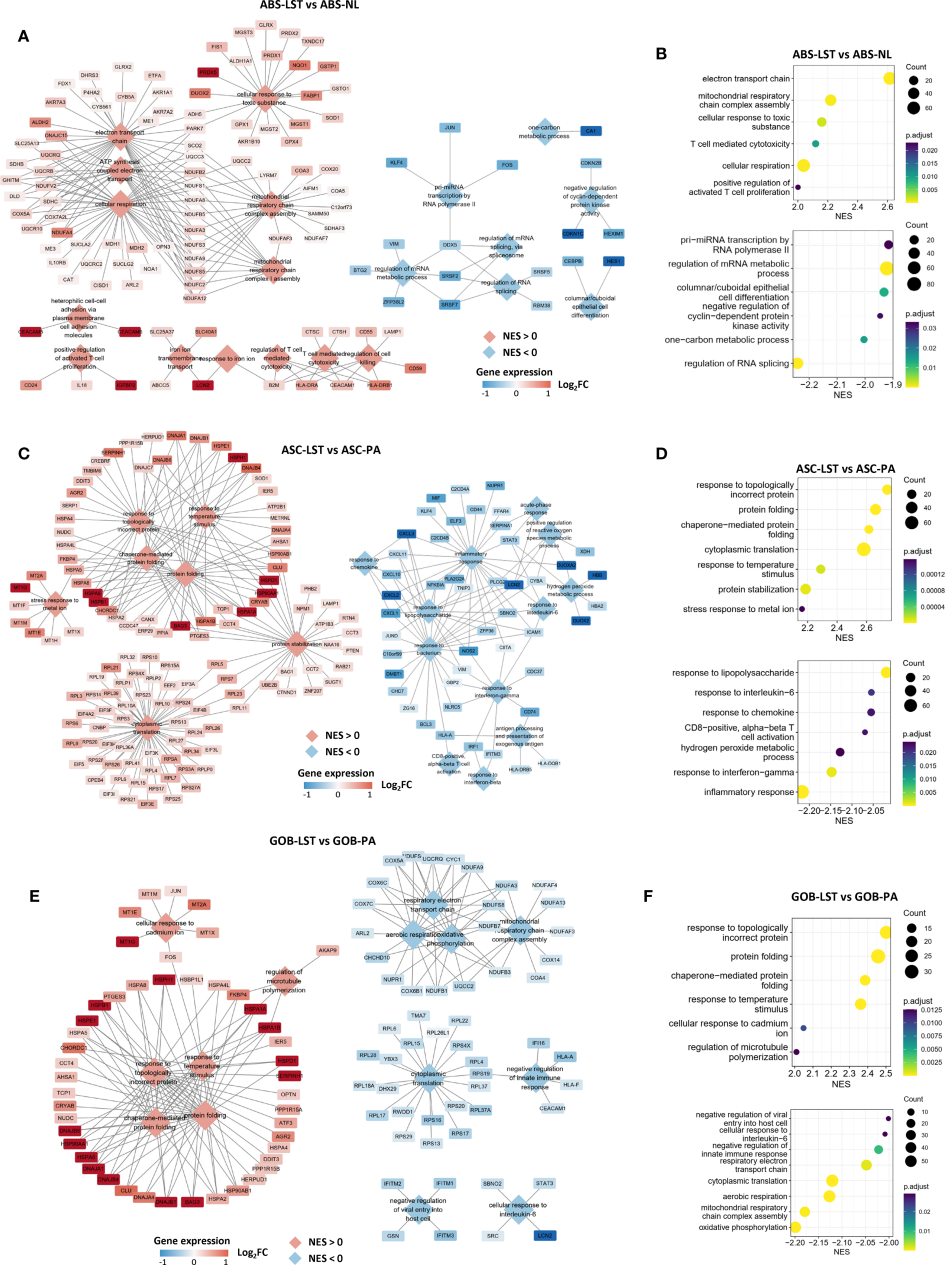
Differences in gene expression of shared epithelial subpopulations across different sample types. **(A, B)** GSEA outcomes comparing the ABS-LST and ABL-NL populations. The network diagram illustrates the gene sets obtained from the enrichment analysis and the genes they contain. Diamonds represent gene sets, while rectangles represent individual genes. The same applies below. **(C, D)** GSEA outcomes comparing the ASC-LST and ASC-PA populations. **(E, F)** GSEA outcomes comparing the GOB-LST and GOB-PA populations.

In addition to the specific cell populations, we also identified shared cell populations in LST, PA, and NL samples. Notably, these common cell populations exhibited distinct variations when analyzed across different sample types. Firstly, ABS found in LST samples (ABS-LST) exhibited differences from those in NL samples (ABS-NL). Specifically, LST samples derived from patients P1 and P5 were paired with corresponding NL samples. Utilizing UMAP analysis based on RNA space, we observed marked transcriptional disparities between ABS-LST and ABS-NL, with these populations forming two separate clusters (**Supplementary Figures 5A, B**). Conversely, the two NL samples displayed a high degree of similarity in RNA space, effectively mitigating concerns of batch effects (**Supplementary Figure 5C**). Differential gene expression and enrichment analysis further revealed that ABS-LST demonstrated significant upregulation of genes associated with aerobic respiration, as compared to ABS-NL (**Figures 3A, B**, and **Supplementary Figure 5D**). Additionally, genes involved in responses to oxidative stress and toxicants were also found to be upregulated.

Moreover, gene expression discrepancies were evident between ASC in LST samples (ASC-LST) and ASC in PA samples (ASC-PA). Our findings indicated that ASC-LST showed notable upregulation of multiple genes related to protein homeostasis maintenance, encompassing a range of chaperones (**Figures 3C, D**, and **Supplementary Figure 5E**). Furthermore, metallothioneins play a crucial role in intracellular metal ion metabolism and redox homeostasis (35). Genes encoding metallothioneins were also found to be upregulated (**Figure 3C**). Additionally, genes associated with ribosomal function were upregulated, suggesting enhanced protein synthesis in ASC-LST (**Figure 3C**). Conversely, genes linked to immune responses were downregulated in ASC-LST (**Figure 3C**).

Goblet cells (GOB) represent a substantial proportion of cells in NL, PA, and LST samples. When comparing GOB in LST (GOB-LST) to GOB in PA (GOB-PA), we observed significant upregulation of various chaperones and metallothionein-encoding genes in GOB-LST (**Figures 3E, F** and **Supplementary Figure 5F**). Pathways related to respiratory metabolism and immune responses were downregulated. It is noteworthy that, upon scoring GOB in each sample group using established signatures of ASC (**Supplementary Table S2**), GOB-LST and GOB-PA exhibited significantly higher WNT pathway activity and ASC signature scores than GOB-NL (**Supplementary Figure 5G**). Consequently, it can be inferred that GOB in diseased samples do not constitute normal goblet cells.

### 3.4 Precancerous LST-G shares transcriptional similarities with colorectal cancer

To investigate the relationship between the gene expression characteristics of LST-G and its progression to carcinoma, we utilized our LST dataset along with publicly available datasets pertaining to CRC and the precancerous lesions. Firstly, we found that metaplastic differentiation was absent in the earliest AD lesions. In the COLON MAP dataset, non-advanced AD samples exhibited minimal presence of SSC. However, as previously stated, a discernible number of SSCs were present in LST (**Figure 1E**). It is noteworthy that MSS CRC is generally considered to arise from AD lesions, whereas microsatellite instability (MSI) CRC originates from SER. Consistently, our analysis of multiple single-cell datasets demonstrated that the epithelial population of MSI CRC had markedly higher SSC signature scores and metaplasia score than that of MSS (**Supplementary Figure 6A**). Within MSS CRCs, however, there was considerable heterogeneity in the expression of genes associated with metaplastic differentiation and SSC populations. In some MSS samples, the expression levels of these genes were comparable to or even exceeded those in MSI CRCs (**Supplementary Figures 6B–D**). Analogously, in adenomatous LST-G, the ASC population also exhibited elevated expression of genes related to metaplasia, with some genes being significantly more expressed than in ASC-PA (**Supplementary Figure 6E, F**). These results indicated that adenomatous LST-G exhibited metaplastic differentiation characteristics akin to that observed in MSS CRC.

**Figure 4:**
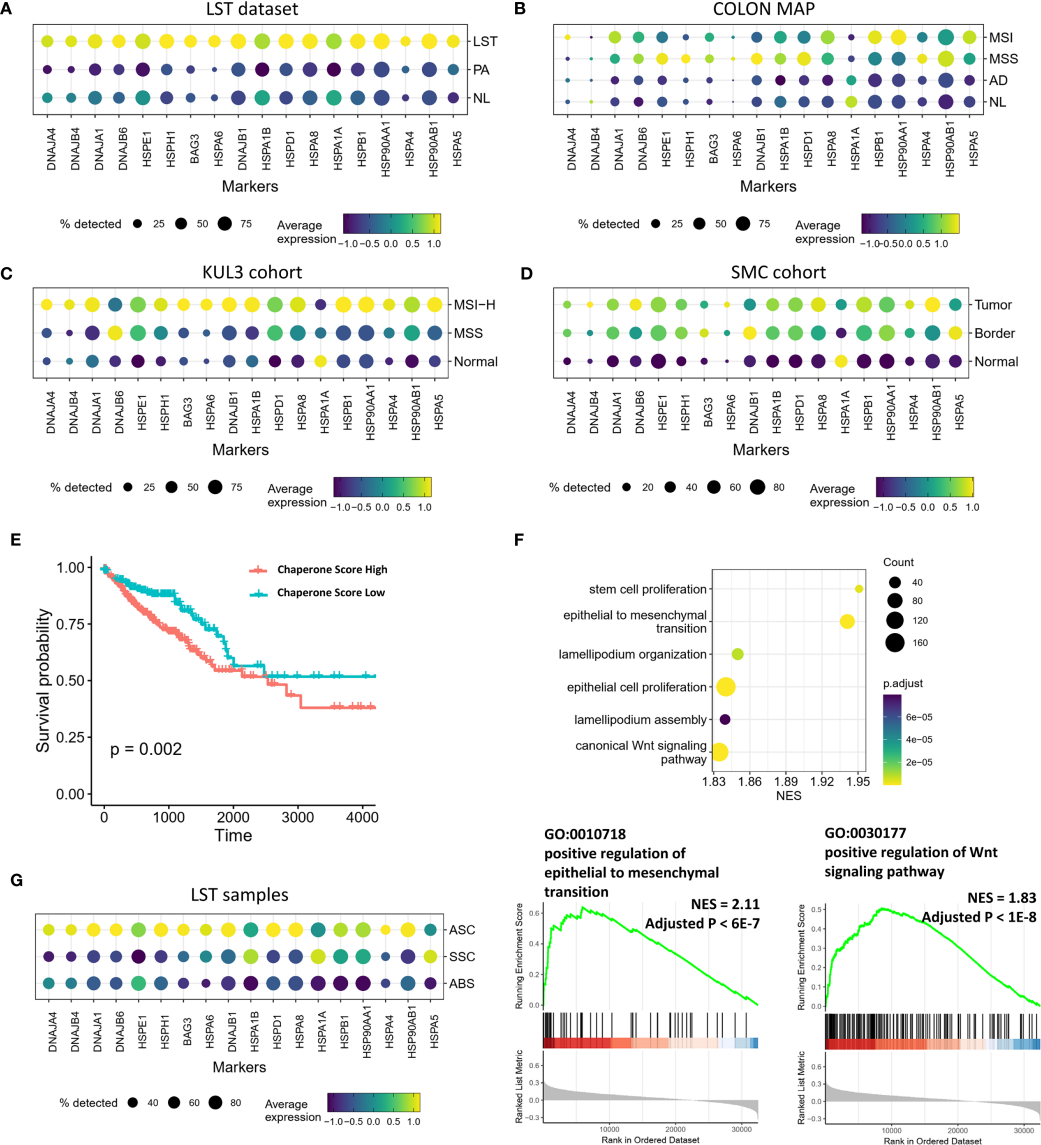
Transcriptional profiles of chaperones in precancerous lesions and CRCs. **(A–D)** Expression of chaperone in precancerous and cancerous groups from multiple datasets. **(E)** Kaplan-Meier survival analysis stratified by chaperone signature score. **(F)** GSEA outcomes illustrating the pathways associated with the chaperone signature score. **(G)** Expression of chaperones in ASC, SSC, and ABS populations in LSTs.

Beyond metaplastic differentiation, disparities in chaperone expression levels were also evident between CRC and precancerous lesions. As previously mentioned, adenomatous LST-G displayed significantly higher expression of chaperones and related cofactors compared to PA and NL samples (**Figure 4A**). However, the COLON MAP dataset indicated that the earliest AD lesions did not exhibit significant differences in the expression of these genes compared to NL samples (**Figure 4B**). By contrast, in both MSS-type and MSI-type CRCs, the expression levels of these molecules were significantly higher than in NL samples (**Figure 4B**). Comparable results were observed in other datasets, with the CRC group in the SMC and KUL3 datasets showing significantly higher expression of chaperones and related factors compared to the normal group (**Figures 4C, D**). Overall, the expression levels of these molecules increased with tumor progression and malignancy. We selected the most significantly upregulated chaperones in LST compared to PA (**Supplementary Table S2**) and utilized them as a signature to score the TCGA data. The results indicated that the high-score group had a poorer prognosis compared to the low-score group (**Figure 4E**). GSEA based on the correlation between other genes and the obtained scores revealed that the score was positively correlated with stem cell proliferation, EMT, lamellipodia function, and the WNT signaling pathway (**Figure 4F**), suggesting that these chaperones may collectively exert tumor-promoting effects. Notably, in LST samples, the SSC and ABS populations, which were more differentiated, showed significantly lower chaperone expression levels compared to the ASC population with higher stemness, indicating that chaperones may contribute to the maintenance of stemness (**Figure 4G**).

Furthermore, as mentioned above, ASC-LST exhibited significantly upregulated expression of several genes encoding metallothioneins, particularly MT1E, MT1G, MT1M, and MT2A, compared to ASC-PA. In fact, these genes were more highly expressed in the differentiated ABS population than in other epithelial populations (**Supplementary Figure 7A**). In the TCGA data, the expression levels of these genes were positively correlated with ABS signature scores and SSC signature scores overall (**Supplementary Figure 7B**). These findings suggest that the expression of these metallothionein genes is related to the level of differentiation in both LST and CRC. The differentiation state of epithelial cells may be associated with pathways involved in metal ion metabolism and the oxidative stress response.

Overall, in comparison to the earliest stage of AD lesions, adenomatous LST-G displayed gene expression patterns that were more akin to CRC. The upregulation of genes related to metaplasia, molecular chaperones, and metallothioneins indicates the progression of LST-G towards CRC.

### 3.5 Cell differentiation state in LSTs is associated with Arp2/3 complex-mediated actin polymerization

**Figure 5:**
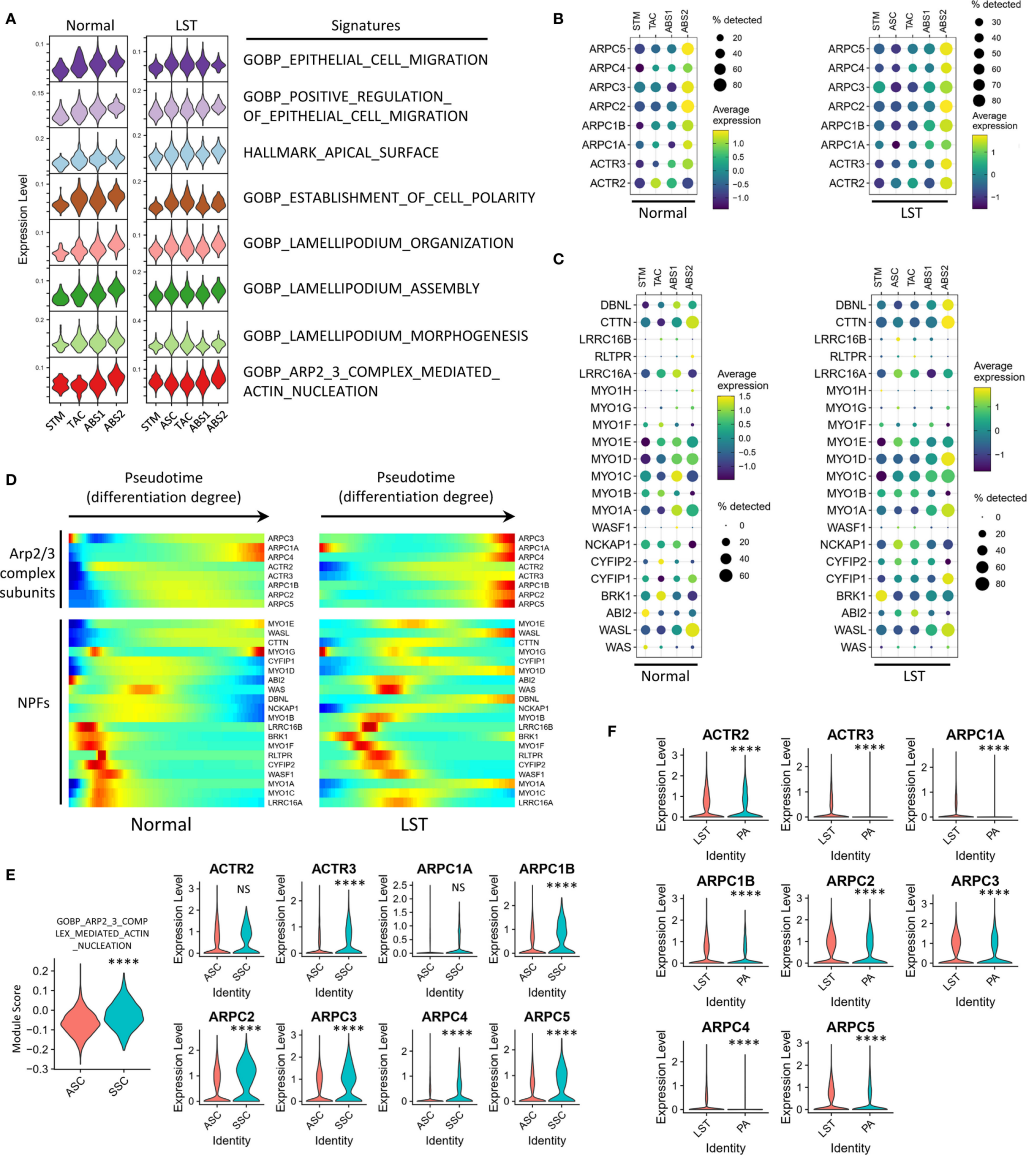
Correlation between cell differentiation state and Arp2/3 complex-mediated actin polymerization in LSTs. **(A)** Assessment of pathway activities related to cell morphology and motility across epithelial populations in NL and LST samples. **(B)** Expression of genes encoding Arp2/3 complex subunit proteins across epithelial populations in NL and LST samples. **(C)** Expression of nucleation-promoting factors (NPFs) in NL and LST samples. **(D)** Pseudotime analysis illustrating the trends in NPFs and Arp2/3 subunits expression in NL and LST samples. **(E)** Expression of Arp2/3 pathway and genes encoding Arp2/3 complex subunits in ASC and SSC populations in LST dataset. **(F)** Comparison of the expression of Arp2/3 complex subunits between all epithelial cells in LST and PA groups. Statistical significance levels are denoted as follows: *P < 0.05; **P < 0.01; ***P < 0.001; ****P < 0.0001.

In addition to the transcriptional features associated with tumor progression, we also attempted to elucidate the mechanisms driving LST morphogenesis using the single-cell gene expression profiles. Given the well-documented movement mechanisms of normal intestinal epithelial cells (13), we initially sought to characterize the gene expression patterns corresponding to these normal cells’ motility patterns. Single-cell transcriptome analysis indicated that, in both NL samples from the COLON MAP dataset and our LST dataset, the gene signatures related to Arp2/3 complex-mediated actin nucleation and lamellipodium assembly were progressively upregulated as STM population differentiated into ABS (**Figure 5A**, **Supplementary Figure 8A** and **Supplementary Table S3**). Consistent with this, the expression of genes encoding Arp2/3 complex subunits increased with cell differentiation (**Figure 5B** and **Supplementary Figure 8B**). It is important to highlight that the Arp2/3 complex possesses little biochemical activity on its own. Nucleation-promoting factor (NPF) proteins activate the Arp2/3 complex, thereby initiating the formation of new (daughter) filament branches from existing (mother) filaments in a y-branch configuration. Single-cell transcriptome data revealed that, while not all NPFs were upregulated with cell differentiation, the expression levels of multiple NPFs were tightly associated with the degree of differentiation. The trends in NPF expression were largely consistent between the COLON MAP dataset and our LST dataset (**Figure 5C** and **Supplementary Figure 8C**). Overall, the expression levels of genes related to the collective migration of normal epithelial cells were strongly linked to the degree of differentiation, suggesting that changes in the expression levels of these genes contribute to collective cell motility.

Notably, SERs often appear as flat lesions, while ADs tend to be protruded. Considering this, we further analyzed the expression of the aforementioned genes in typical SSCs from the SER samples in COLON MAP dataset. The results showed that the scores for the “GOBP_ARP2_3_COMPLEX_MEDIATED_ACTIN_NUCLEATION” signature were not significantly different between SSCs and ABS2 cells, but were significantly higher than those in less differentiated cell populations such as ASCs (**Supplementary Figure 8D**). Similarly, although SSCs had lower scores for the lamellipodium-associated signatures than ABS2 cells, they were still significantly higher than those in ASCs (**Supplementary Figures 8E, F**). Furthermore, the expression levels of genes encoding Arp2/3 complex subunits in SSCs were higher than those in ASCs (**Supplementary Figure 8G**).

Next, we analyzed the expression of these genes in SSCs and ABS cells from LST samples. Briefly, LST samples demonstrated similar expression patterns of Arp2/3 subunits and NPFs to those observed in normal mucosa and SERs. The results exhibited that, in LST samples, both the Arp2/3 signature scores and the expression levels of Arp2/3 subunits were significantly higher in ABS2 cells than in ASCs (**Figures 5A, B**). The expression patterns of NPFs in cells with different differentiation levels in LST were similar to those in NL samples (**Figure 5C**). Pseudotime analysis also supported these findings (**Figure 5D**). Moreover, SSCs in LST exhibited similar properties, with significantly higher Arp2/3 signature scores and expression levels of most Arp2/3 subunits compared to ASCs (**Figure 5E**). Additionally, a comparison of all epithelial cells in LST and PA revealed higher expression of Arp2/3 subunits in LST (**Figure 5F**).

In summary, the expression patterns of Arp2/3 subunits and NPFs in LST samples were similar to those in NL and SER samples. Notably, using SCENIC, we inferred the regulatory relationships between Arp2/3 complex subunits and differentiation-related transcription factors. The results indicated that most regulons capable of regulating the expression of Arp2/3 subunit genes were key transcription factors highly expressed in SSCs and ABS cells (**Supplementary Figure 8H**). This suggests that the gene expression of Arp2/3 complex subunits is tightly coupled with the transcriptional regulation of the differentiation process.

## 4 Discussion

Up to now, the evolutionary trajectory from LST-G to carcinoma, as well as the morphogenetic mechanisms of LST-G, remain unclear. In this study, we utilized single-cell transcriptome technology to analyze the gene expression patterns of LST-G and identified a cell population undergoing metaplastic differentiation. Further analysis revealed that this metaplastic differentiation was closely associated with both the progression of LST-G to carcinoma and the morphogenesis of LST-G. On the one hand, LST-G exhibited high levels of metaplastic differentiation, as well as increased expression of chaperones and metallothioneins, indicating similarities with CRC. On the other hand, the expression pattern of cell motility-related genes in LST-G resembled that of normal mucosa and SER. As the level of metaplastic differentiation increased, the expression of Arp2/3 complex subunits was upregulated, indicating the potential for lateral movement along the basement membrane.

High-frequency KRAS mutations constitute the most prominent and well-recognized genomic feature of adenomatous LST-G (5–7). Prior research has indicated that ADs exhibit a high frequency of APC mutations, whereas KRAS mutations accumulate as ADs progress (9). In addition, SERs, which exhibit a high level of metaplastic differentiation, are characterized by a high frequency of BRAF mutations and a low frequency of KRAS mutations (9). However, the relationship between high-frequency KRAS mutations and metaplastic differentiation remains unclear in colorectal precancerous lesions. Notably, studies of lesions at the CRC stage have documented a correlation between KRAS mutations and metaplastic differentiation. Building upon the consensus molecular subtypes (CMS) of colorectal cancer (36), recent single-cell studies have introduced the intrinsic CMS (iCMS) classification (37). Based on the properties of epithelial cell populations, CRC can be categorized into iCMS2 and iCMS3 subtypes. Among the subtypes, iCMS3 resembles SSCs, displaying higher MAPK pathway activity and a higher level of metaplastic differentiation. Significantly, iCMS3 CRC is marked by high frequencies of KRAS and BRAF mutations, both of which are key nodes in the MAPK signaling pathway. This suggests that metaplastic differentiation may depend on the activation of the MAPK pathway. Interestingly, adenomatous LST-G also displays a similar pattern. A crucial observation is the KRAS-dependent signature exhibited by the SSC population in LST-G, implying that KRAS mutations may contribute to the survival of these cells. Consistent with this, the SSC population in LST-G demonstrates elevated expression of EGFR and ERBB2, which also occupy upstream positions in the MAPK signaling, potentially synergizing with KRAS mutations to promote the survival of metaplastic cells. Therefore, metaplastic differentiation in LST-G may be a consequence mediated by KRAS mutations.

Adenomatous LST-G exhibits both high-frequency KRAS mutations and metaplastic differentiation, suggesting a potential trajectory for adenomatous LST-G to develop into carcinoma. SSC and ABS populations in LST-G exhibit significantly higher expression of MDK, which may possess certain tumor-promoting effects (38). Furthermore, the elevated levels of chaperones and metallothioneins in LST-G also suggest its progression towards CRC. Studies have shown that certain stress factors may upregulate molecules such as chaperones and metallothioneins (35, 39). Therefore, the upregulation of these genes in LST-G indicates a more hostile microenvironment than the earliest stages of AD, resembling conditions within carcinoma-stage lesions. Notably, these factors can also influence the differentiation state of cells. Prior research has shown that MT1G can suppress cell stemness and enhance cell differentiation (40). Additionally, a harsh microenvironment may damage epithelial cells, leading to metaplasia. These factors, along with the accumulation of KRAS mutations, may collectively affect the level of metaplastic differentiation. As noted earlier, MSS CRC typically arises from AD lesions, whereas MSI CRC originates from SER. Notably, although iCMS3 exhibits a higher degree of metaplastic differentiation and is closely associated with SER, iCMS3 CRC is not exclusive to the MSI type (37). A significant proportion of iCMS3 CRC is MSS and characterized by a higher frequency of KRAS mutations. Conversely, KRAS mutation frequency is lower in MSI CRC. Further analysis reveals that the frequency of KRAS G12/13 mutations in iCMS3-MSS CRC is significantly higher than that in iCMS2-MSS and iCMS3-MSI CRCs (37). This suggests that KRAS mutations may mediate the metaplastic differentiation in MSS CRC. Similarly, LST-G also exhibits a higher frequency of KRAS mutations and a higher level of metaplastic differentiation. Based on these observations, we hypothesize that adenomatous LST-G is a likely precursor of iCMS3-MSS CRC.

Beyond the relationship between metaplastic differentiation and tumor evolution, our findings also suggest that cell differentiation influences cell morphology, adhesion, intercellular connections, and motility in LST-G. In LST-G samples, as the level of differentiation increases, a series of cell-cell interactions related to adhesion and junction are strengthened, indicating that cells tend to be confined to a single layer of columnar epithelium and adhere to the basement membrane. Furthermore, the upregulation of Arp2/3 subunits, which are associated with collective cell migration, suggests that cells have acquired an enhanced ability to move along the basement membrane. This situation is analogous to that of normal mucosal epithelium. It is plausible that if cells at the LST lesion edges expand into normal mucosa regions, collective migration along the basement membrane may represent a potential mode of spreading. Recent studies have demonstrated that most regions of the normal mucosal single-layer columnar epithelium are under tension rather than compression (13, 41). If the normal mucosal epithelium at the lesion edges is similarly tensioned, then the expansion of lesion cells into normal regions would primarily necessitate overcoming friction with the basement membrane, rather than resistance from normal cells in the direction of movement. The formation of lamellipodia-like structures mediated by Arp2/3 complex at the basement membrane may facilitate overcoming this friction and promote lateral movement. This can be considered as a candidate mechanism for the morphogenesis of LSTs.

Clinically, our findings can help refine risk stratification: LST-Gs with a high proportion of SSCs or elevated expression of SSC-related markers (e.g., metaplastic signature genes, Arp2/3 complex subunits) may be classified as “high-risk” precancerous lesions, warranting more aggressive surveillance or early intervention (e.g., complete endoscopic resection with enhanced margin assessment). In contrast, PAs lacking such SSCs may be managed with standard follow-up protocols, reducing unnecessary medical interventions. In addition, targeting the Arp2/3 complex could potentially reduce the lateral invasiveness of LST-Gs, improving the completeness of endoscopic resection and reducing recurrence rates. For lesions with high Arp2/3 expression, pre-resection assessment of lateral extension (e.g., via advanced imaging modalities like endoscopic ultrasound) or adjuvant therapies (e.g., local ablation) may be considered to prevent residual tumor spread. Moreover, our observations support the potential of these molecules as biomarkers: Serum or tissue levels of chaperones or metallothioneins could serve as non-invasive indicators for monitoring LST-G progression to CRC. Additionally, our findings support the development of personalized follow-up strategies: patients with LST-Gs positive for SSC signatures or high chaperone/metallothionein expression may require more frequent colonoscopic surveillance (e.g., every 6–12 months) compared to those with low-risk lesions.

In conclusion, the differentiation state of cells within LST-G exhibits a close correlation with their diverse characteristics. Metaplastic differentiation, as a prominent feature at the transcriptional level, demonstrates robust associations with the genomic features, morphogenesis, and tumor progression of LST-G. Our research has contributed to an enhanced comprehension of the molecular features and evolutionary dynamics of colorectal precancerous lesions. Our exploration into the evolutionary pathways and morphological mechanisms of LST-G sheds light on its malignant progression and microenvironment, providing a basis for precision research into pathogenesis and prevention of CRC.

Our study has several limitations that warrant consideration. First, while single-cell RNA sequencing enabled detailed dissection of LST-G cellular heterogeneity, it lacks spatial resolution, limiting direct mapping of key transcriptional signatures—such as Arp2/3 upregulation or the distribution of SSCs and ABSs—to anatomical regions critical for lateral spread, particularly the tumor-normal interface. This hinders precise linkage between molecular features and the spatial dynamics of LST-G morphogenesis. Second, the relatively small sample size, stemming from the rarity of LST-Gs and strict inclusion criteria, constrains statistical power. Additionally, sample stratification presents limitations: LST-G samples included both TA and TVA subtypes, while PA samples were exclusively TA. Though this distribution reflects clinical reality (LST-G more frequently presents as TVA), the small sample size limits robust subtype-stratified analyses.

Despite these constraints, our findings are supported by multiple lines of evidence. Indirect spatial inferences align with known patterns: differentiated SSCs and ABSs in LST-G share Arp2/3-mediated motility gene expression with normal intestinal epithelium, suggesting conserved localization to active lateral expansion areas; their enhanced adhesion molecules and immune communication fit the tumor-normal interface; and integration with the COLON MAP dataset shows SERs (flat, like LST-G) position SSCs in lateral extension regions, analogous to LST-G’s SSCs. Controls (PA, NL) were carefully selected, including paired NL from LST-G patients to reduce variability, and consistent biological signals across LST-Gs (e.g., metaplastic SSCs, Arp2/3 upregulation in differentiated cells), validation via COLON MAP integration, and rigorous methods minimize sample size impacts.

Future work will address these limitations by incorporating spatial transcriptomics and multiplex immunofluorescence to map SSCs, ABSs, and Arp2/3 at the tumor-normal interface, alongside expanded sample cohorts for subtype-specific analyses. Biophysical methodologies will further elucidate cell motility patterns, refining our understanding of LST-G morphogenesis and progression.

Furthermore, a deeper investigation is warranted to elucidate the details of LST-G progression to CRC, particularly with regard to the transcriptional disparities and similarities between early cancer and adenoma tissues adjacent to cancer in LST-G. Additionally, to unravel the morphogenetic mechanisms underlying LST-G, a more comprehensive analysis incorporating biophysical methodologies is necessary to elucidate the motility patterns of cells in LST. The application of spatial transcriptomics and other advanced techniques, which incorporate spatial dimensions into gene expression analysis, may further facilitate the exploration of these issues.

## Data Availability

The original contributions presented in the study are publicly available. This data can be found here: https://doi.org/10.6084/m9.figshare.29925404. Further inquiries can be directed to the corresponding authors.
