# Supplementary material for: Single-cell RNA profiling of colorectal granular-type laterally spreading tumor uncovers progression trajectory toward carcinoma and transcriptional signatures favoring lateral morphogenesis
Source: Front Oncol. 2025 Sep 12;15:1552841. doi: 10.3389/fonc.2025.1552841 (PMC12463592; doi:10.3389/fonc.2025.1552841)
Supplement: Supplementary file 2 [file DataSheet1.pdf]

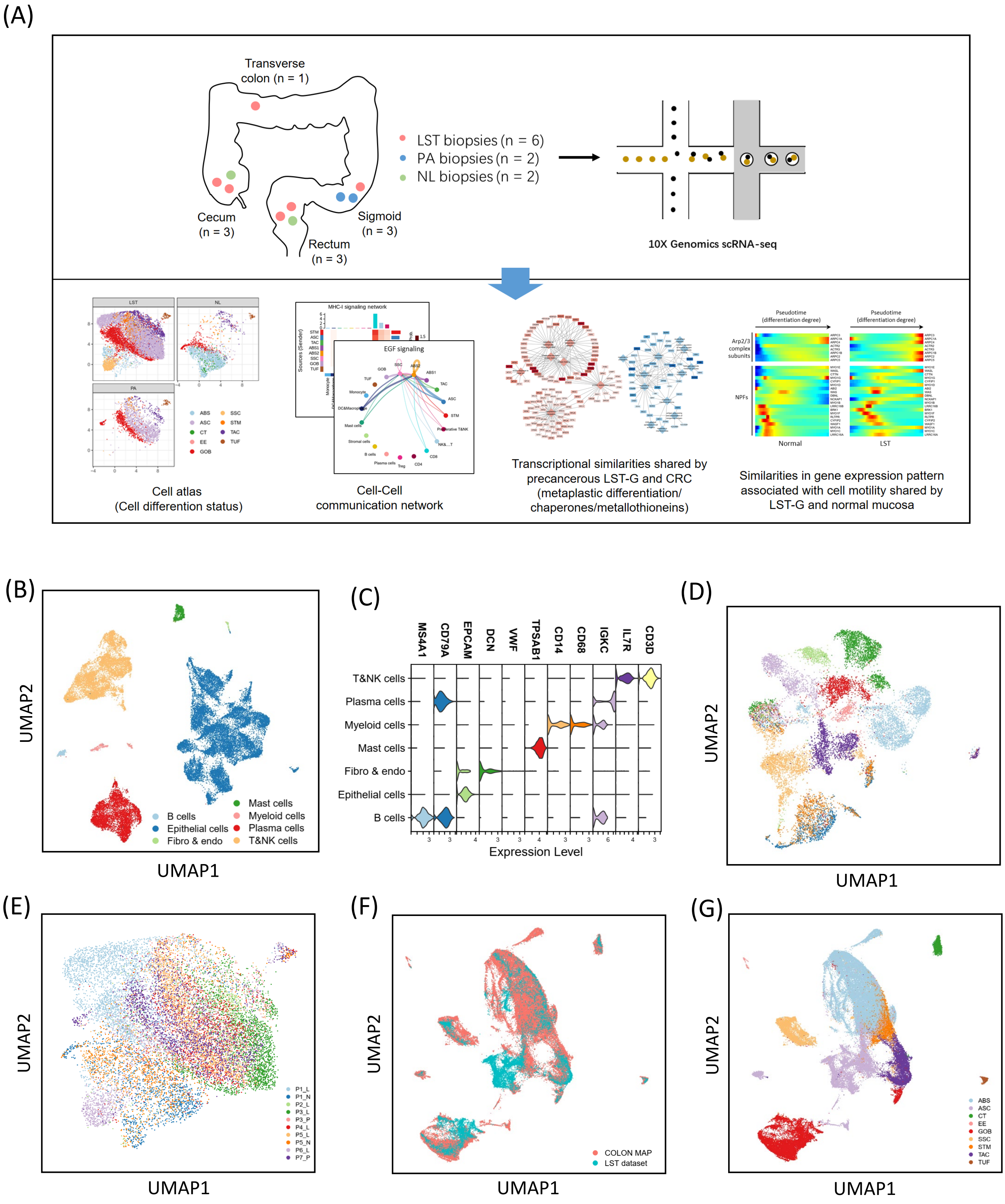

Figure S1. UMAP based on regulon space and integration analysis with COLON MAP dataset
(A) Schematic representation of the single-cell analysis workflow.
(B) UMAP plot illustrating the primary cell lineages.
(C) Expression profiles of key marker genes within distinct primary cell lineages
(D) UMAP plot based on RNA space illustrating polyp-specific effects. Each color represents a single-cell RNA sample.
(E) UMAP based on regulon space effectively adjusted for polyp-specific effects.
(F, G) UMAP plot based on integrated feature space illustrating the merged datasets and the epithelial cell subpopulations.

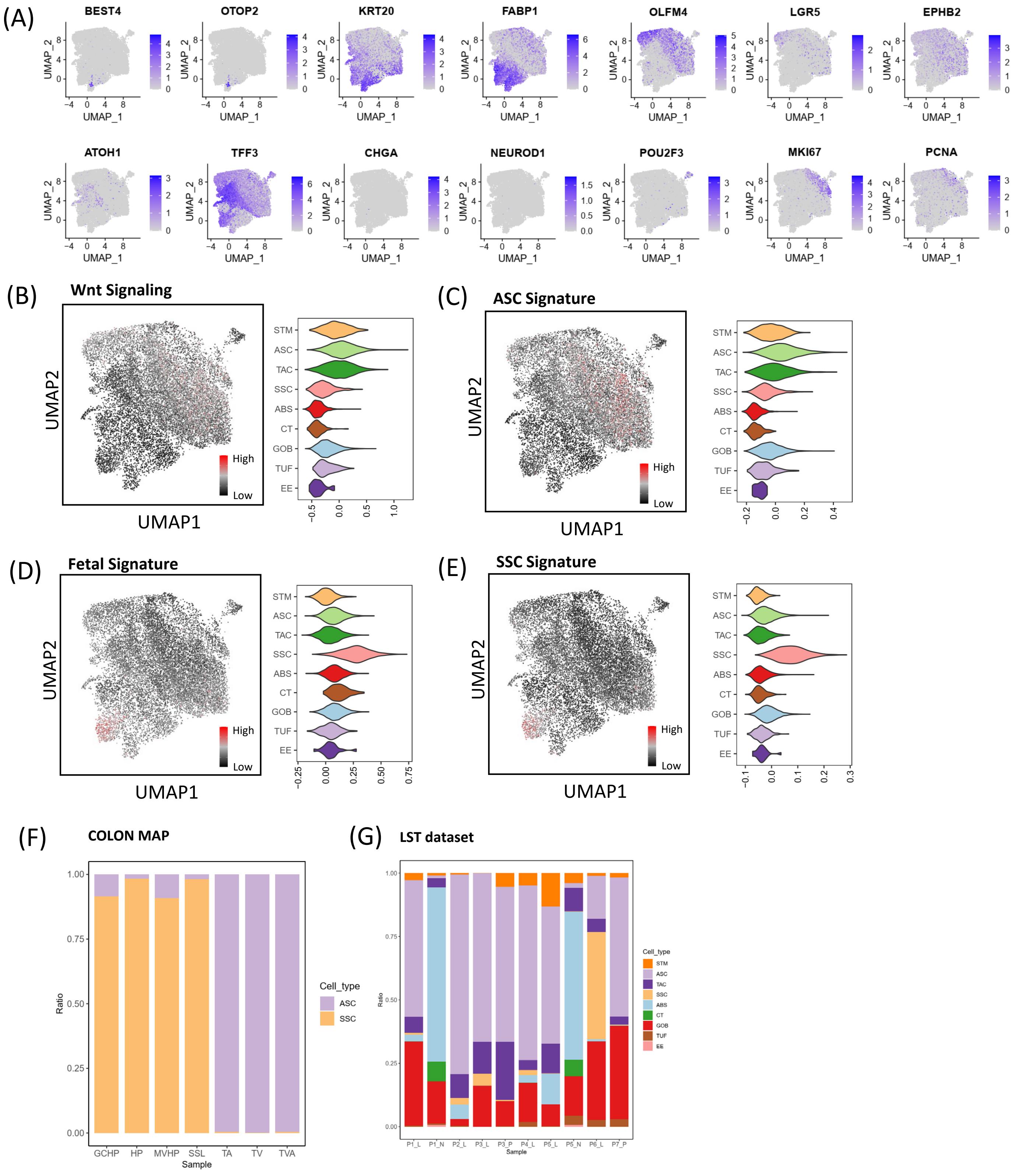

Figure S2. Gene expression features of epithelial subpopulations.
(A) UMAP plot illustrating the expression profiles of key marker genes within distinct epithelial cell subpopulations.
(B, C, D, E) UMAP plot and violin plot illustrating the key signatures of ASC and SSC populations.
(F) Proportions of ASC and SSC populations present in distinct sample groups of COLON MAP dataset. HP, hyperplastic polyp; GCHP, goblet cell-rich HP; microvesicular HP, MVHP; TA, tubular adenoma, TVA, tubulovillous adenoma; TV, villous adenoma.
(G) Proportions of various epithelial subpopulations present in samples of LST dataset.

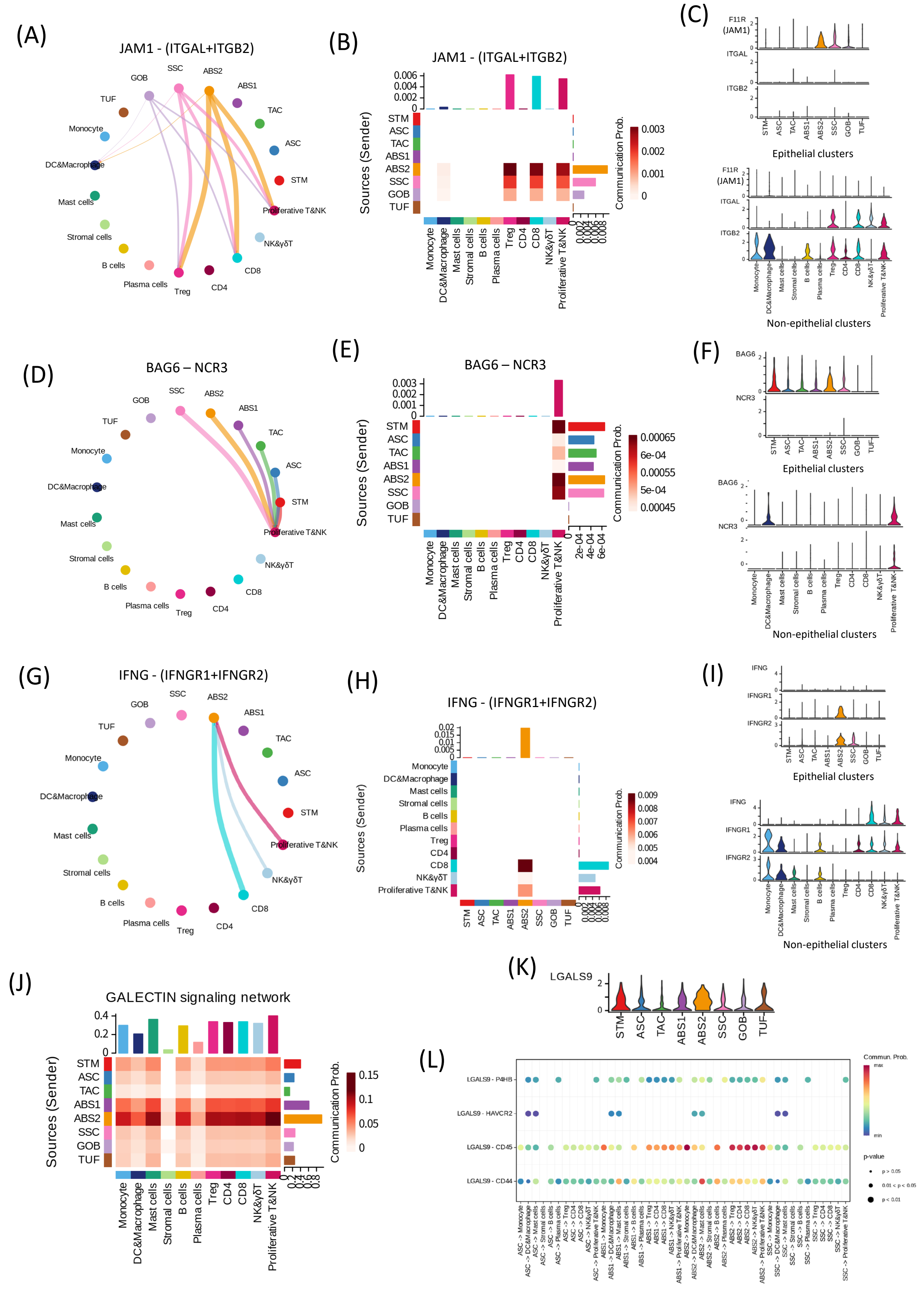

Figure S3. Intercellular interactions associated with immune regulation.
(A, B, C) Intercellular communication status via JAM1-(ITGAL+ITGB2) interaction.
(D, E, F) Intercellular communication status via BAG6-NCR3 interaction.
(G, H, I) Intercellular communication status via IFNG-(IFNGR1+IFNGR2) interaction.
(J, K, L) Intercellular communication status via galectin-9 signaling.

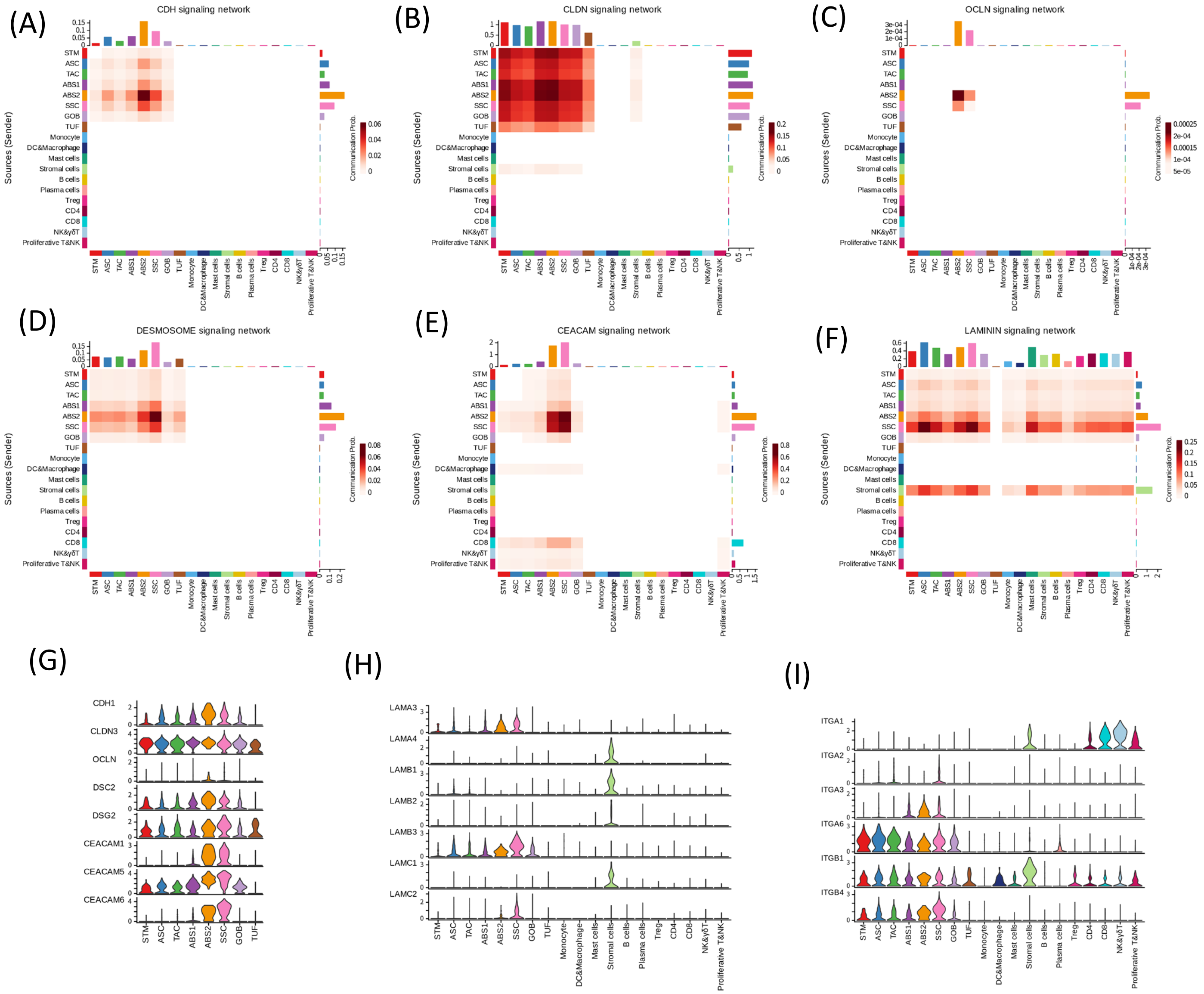

Figure S4. Intercellular interactions associated with cell adhesion and junctions.
(A, B, C, D, E, F) Strength of interactions associated with cell adhesion and junctions.
(G, H, I) Expression of genes associated with cell adhesion and junctions.

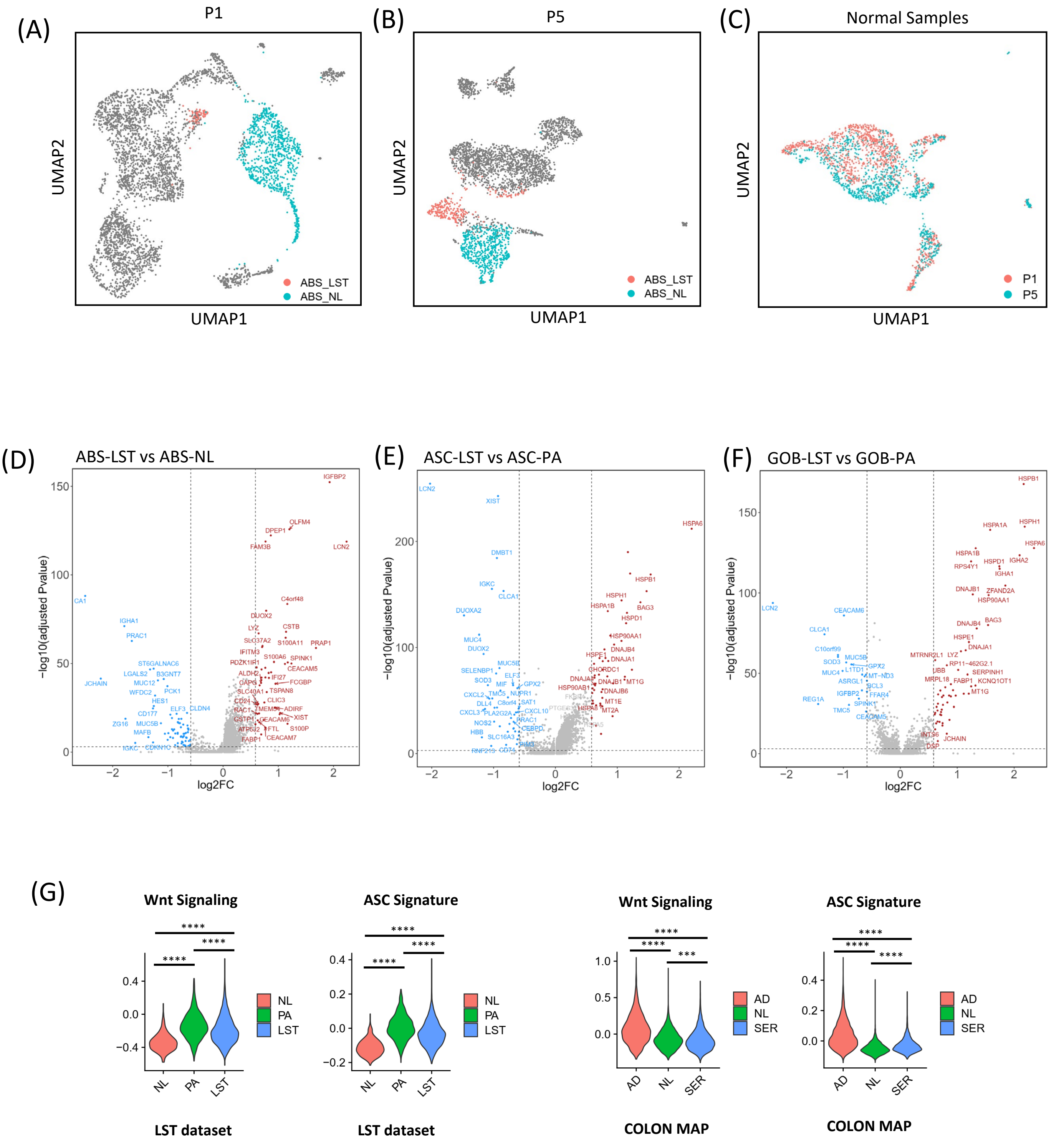

Figure S5. Differences in gene expression of shared epithelial subpopulations

(A, B) UMAP plot based on RNA space illustrating the transcriptional disparities between ABS-LST and ABS-NL.

(C) UMAP plot based on RNA space illustrating the consistency of transcriptional features of NL samples.

(D, E, F) Volcano plot illustrating the differential expressed genes of shared epithelial subpopulations across different sample types.

(G) Scoring GOBs in both LST dataset and COLON MAP dataset using established signatures of ASC and Wnt signaling.

Statistical significance levels are denoted as follows: *, $P < 0.05$; **, $P < 0.01$; ***, $P < 0.001$; ****, $P < 0.0001$.

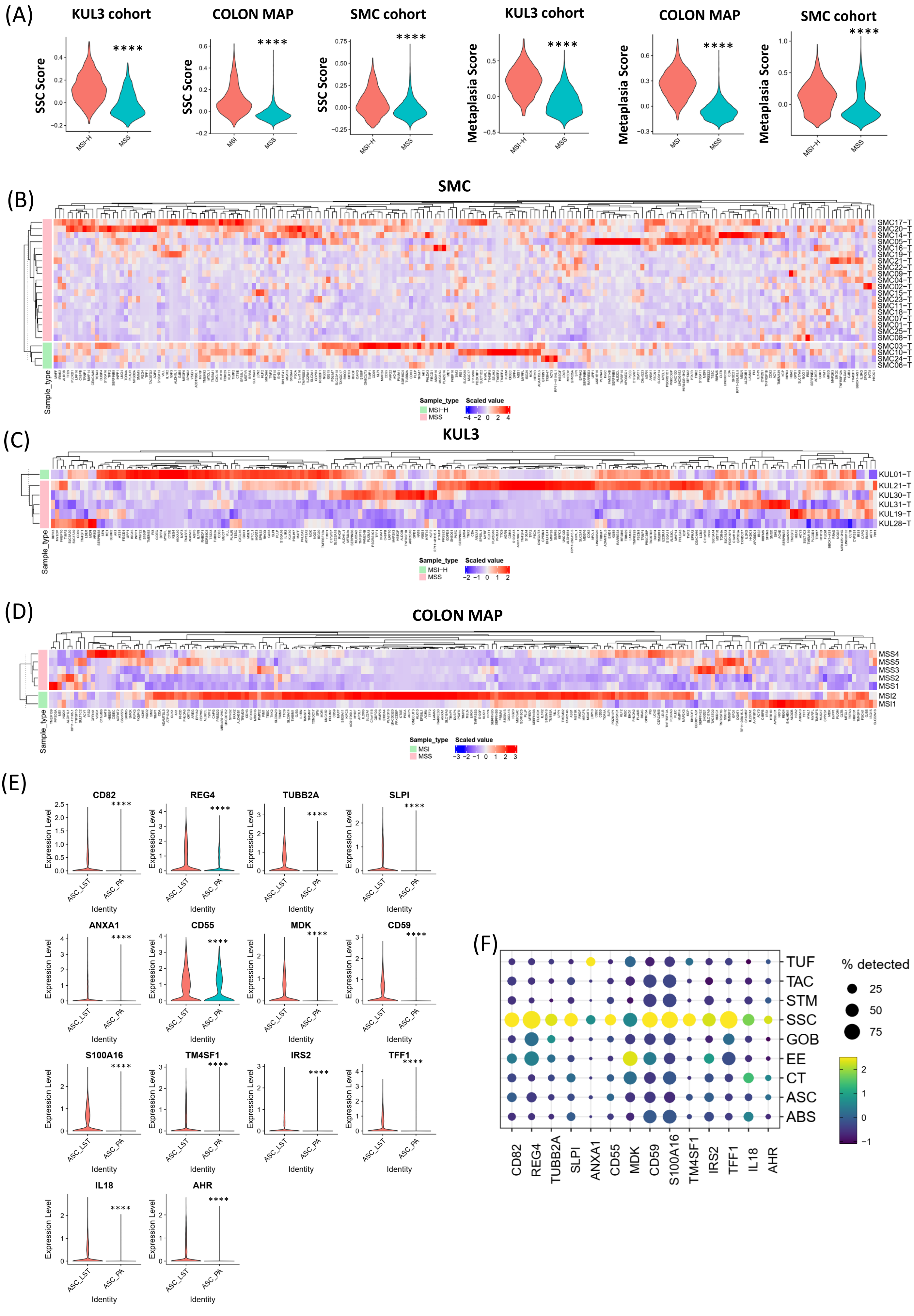
(A)
KUL3 cohort
COLON MAP
SMC cohort
KUL3 cohort
COLON MAP
SMC cohort
SSC Score
Metaplasia Score
****
MSI-H
MSI
MSS
(B)
SMC
SMC17-T
SMC20-T
SMC14-T
SMC05-T
SMC16-T
SMC19-T
SMC21-T
SMC22-T
SMC09-T
SMC04-T
SMC02-T
SMC15-T
SMC23-T
SMC11-T
SMC18-T
SMC07-T
SMC01-T
SMC25-T
SMC08-T
SMC03-T
SMC10-T
SMC24-T
SMC06-T
Sample_type
Scaled value
MSI-H
MSS
(C)
KUL3
KUL01-T
KUL21-T
KUL30-T
KUL31-T
KUL19-T
KUL28-T
Sample_type
Scaled value
MSI-H
MSS
(D)
COLON MAP
MSS4
MSS5
MSS3
MSS2
MSS1
MSI2
MSI1
Sample_type
Scaled value
MSI
MSS
(E)
CD82
REG4
TUBB2A
SLPI
ANXA1
CD55
MDK
CD59
S100A16
TM4SF1
IRS2
TFF1
IL18
AHR
Expression Level
Identity
ASC_LST
ASC_PA
****
(F)
TUF
TAC
STM
SSC
GOB
EE
CT
ASC
ABS
CD82
REG4
TUBB2A
SLPI
ANXA1
CD55
MDK
CD59
S100A16
TM4SF1
IRS2
TFF1
IL18
AHR
% detected
25
50
75

Figure S6. Assessment of metaplastic differentiation in precancerous lesions and CRCs.
(A) Comparisons of the SSC and metaplasia signatures between MSI and MSS CRC in multiple datasets.
(B, C, D) Heatmaps illustrating the expression of genes associated with metaplastic differentiation in MSS and MSI CRCs from multiple datasets.
(E, F) Expression of genes associated with metaplastic differentiation in ASC-LST and ASC-PA. (F) indicates these genes are highly expressed in typical SSCs in SER samples.
Statistical significance levels are denoted as follows: ****, $P < 0.0001$.

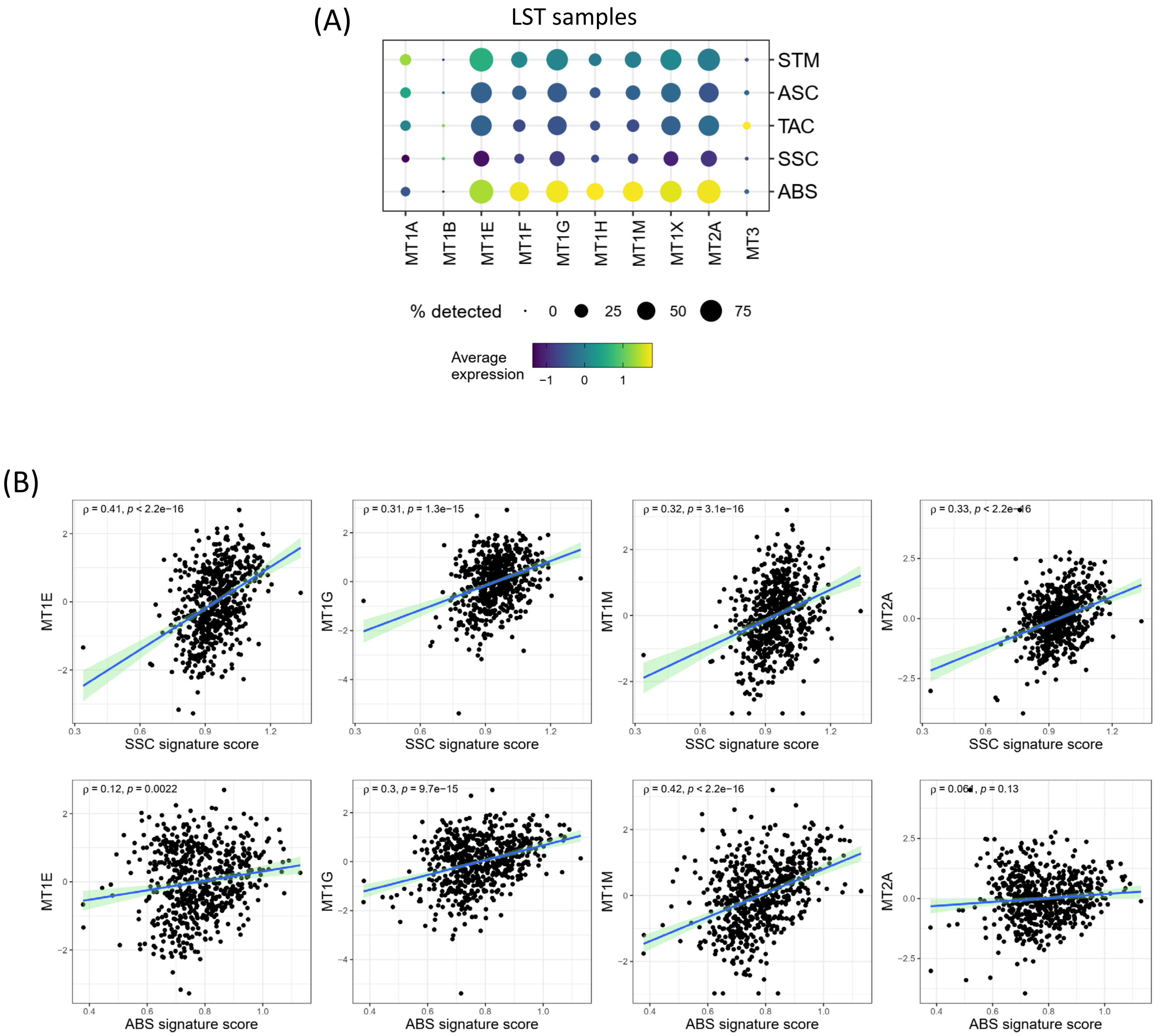

Figure S7. Correlation between cell differentiation state and the expression of metallothioneins (MTs) in LST and CRC samples.
(A) Expression of MTs in epithelial populations in LST samples.
(B) Correlation between cell differentiation scores and the expression of MTs in TCGA cohort.

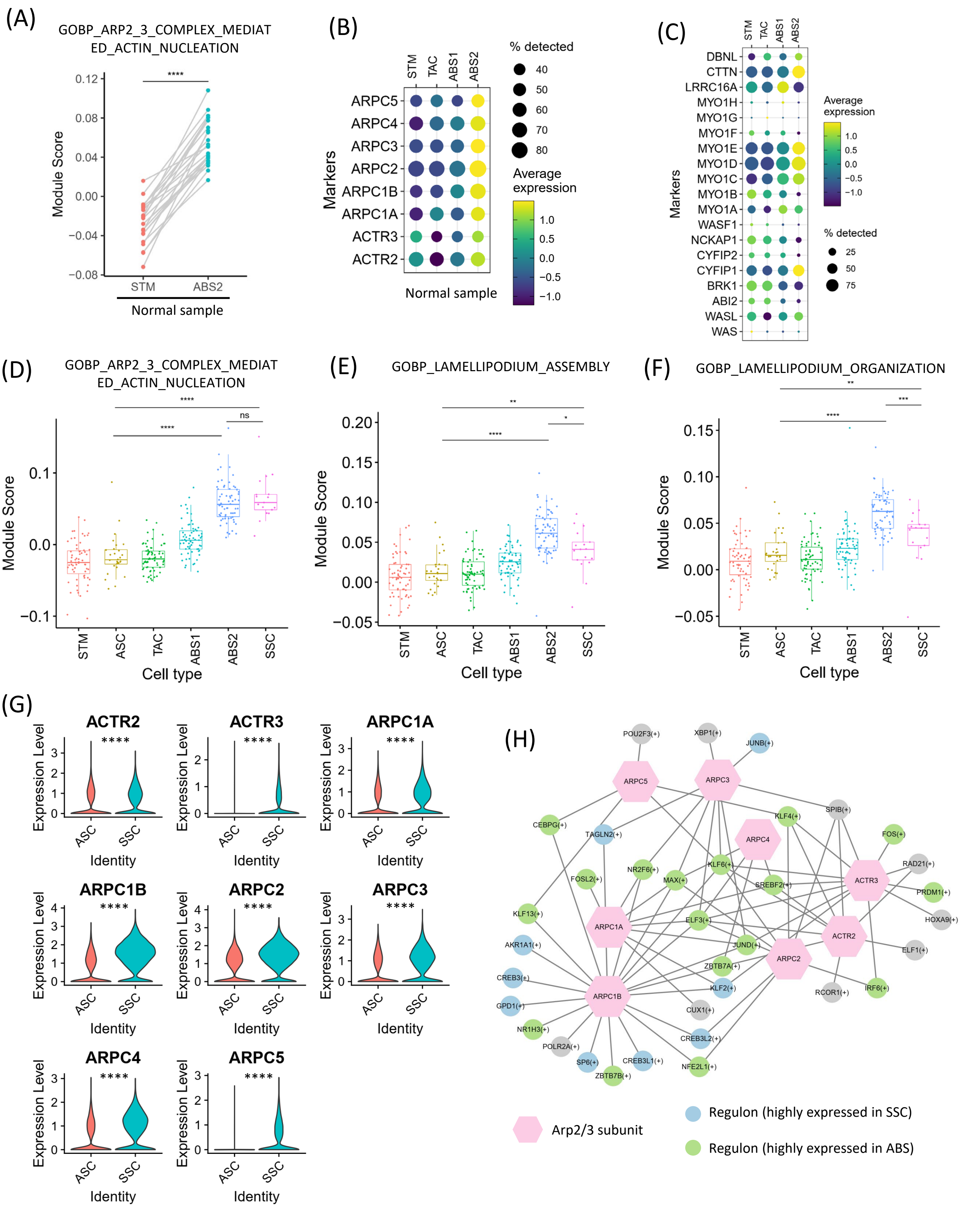
(A)
GOBP_ARP2_3_COMPLEX_MEDIATED_ACTIN_NUCLEATION
Module Score
****
STM
ABS2
Normal sample
(B)
STM TAC ABS1 ABS2
ARPC5
ARPC4
ARPC3
ARPC2
ARPC1B
ARPC1A
ACTR3
ACTR2
Markers
Normal sample
% detected
Average expression
(C)
STM TAC ABS1 ABS2
DBNL
CTTN
LRRC16A
MYO1H
MYO1G
MYO1F
MYO1E
MYO1D
MYO1C
MYO1B
MYO1A
WASF1
NCKAP1
CYFIP2
CYFIP1
BRK1
ABI2
WASL
WAS
Markers
Average expression
% detected
(D)
GOBP_ARP2_3_COMPLEX_MEDIATED_ACTIN_NUCLEATION
Module Score
Cell type
STM ASC TAC ABS1 ABS2 SSC
(E)
GOBP_LAMELLIPODIUM_ASSEMBLY
Module Score
Cell type
STM ASC TAC ABS1 ABS2 SSC
(F)
GOBP_LAMELLIPODIUM_ORGANIZATION
Module Score
Cell type
STM ASC TAC ABS1 ABS2 SSC
(G)
ACTR2
ACTR3
ARPC1A
ARPC1B
ARPC2
ARPC3
ARPC4
ARPC5
Expression Level
Identity
ASC SSC
(H)
ARPC5
ARPC3
ARPC4
ARPC1A
ARPC1B
ARPC2
ACTR3
ACTR2
Arp2/3 subunit
Regulon (highly expressed in SSC)
Regulon (highly expressed in ABS)

Figure S8. Assessment of gene expression related to cell morphology and motility in COLON MAP dataset.
(A) Assessment of Arp2/3 complex pathway activities between STM and ABS2 populations in NL samples. Points represent the average signature score of a cell population in individual specimens.
(B) Expression of genes encoding Arp2/3 complex subunit proteins across epithelial populations in NL samples.
(C) Expression of nucleation-promoting factors (NPFs) in NL samples.
(D, E, F) Assessment of pathway activities related to cell morphology and motility across multiple epithelial populations in COLON MAP dataset. Points represent the average signature score of a cell population in individual specimens.
(G) Expression of genes encoding Arp2/3 complex subunits in ASC and SSC populations in COLON MAP dataset.
(H) Transcriptional regulatory relationships between Arp2/3 complex subunits and key transcription factors.
Statistical significance levels are denoted as follows: *, $P < 0.05$; **, $P < 0.01$; ***, $P < 0.001$; ****, $P < 0.0001$.
